# Supplementary material for: A Novel Cre/lox-Based Genetic Tool for Repeated, Targeted and Markerless Gene Integration in Yarrowia lipolytica
Source: Int J Mol Sci. 2021 Oct 4;22(19):10739. doi: 10.3390/ijms221910739 (PMC8509416; doi:10.3390/ijms221910739)
Supplement: Supplementary file 1 [file ijms-22-10739-s001.zip › ijms-1370457-supplementary final (new).pdf]

Supplementary Material

A Novel Cre/*lox*-Based Genetic Tool for Repeated, Targeted and Markerless Gene Integration in *Yarrowia lipolytica*

Qinghua Zhou †, Liangcheng Jiao †, Wenjuan Li, Zhiming Hu, Yunchong Li, Houjin Zhang, Min Yang, Li Xu * and Yunjun Yan *

Key Laboratory of Molecular Biophysics, The Ministry of Education, College of Life Science and Technology, Huazhong University of Science and Technology, Wuhan 430074, China; qinghuazhou1@126.com (Q.Z.); jiaoliangcheng@gmail.com (L.J.); wenjuanli1@163.com (W.L.); zhiming711@163.com (Z.H.); liyunchong77@hust.edu.cn (Y.L.); hjzhang@hust.edu.cn (H.Z.); ymyangmin@hust.edu.cn (M.Y.)

* Correspondence: xuli@mail.hust.edu.cn (L.X.); yanyunjun@hust.edu.cn (Y.Y.)

† These authors contributed equally to this work.

This PDF file includes:

Tables S1 to S2
Figures S1 to S8

Table S1. Primers and *lox* site used in this study.

Primer/*lox* Site	Sequence (5′-3′)	Annotation
**Primer**		
Pox2-F1	CGCATATGATGCCATCCCACAAGACGAA	PCR for pPOX2
Pox2-R1	GGAATTGTTATCCGCTCACAATTCCGGATCCGGCGTCGTTGCTTGTGTGATTT	PCR for pPOX2
Cre-F1	GGAATTGTGAGCGGATAACAATTCCATGTCCAATTTACTGACCGTACA	PCR for *cre* gene
Cre-R1	AGTTGTAAAGAGTGATAAATAGCCCTAGGCTAATCGCCATCTTCCAGCAGGC	PCR for *cre* gene
lip2t-F1	CTGCTGGAAGATGGCGATTAGCCTAGGGCTATTTATCACTCTTTACAACT	PCR for lip2t-*lox*71, lip2t-*lox*66, lip2t-rc*lox*71
lip2t-R1	GCTAGCACGCGTATAACTTCGTATAATGTATGCTATACGAAGTTATCTCCACC TGTGTCAATCTTC	PCR for lip2t-*lox*71
lip2t-R2	GGAGTCGCATAAGGGAGAGCTCTAGAGTCGACACGCGTTACCGTTCGTATA ATGTATGCTATACGAAGTTATCTCCACCTGTGTCAATCTTC	PCR for lip2t-*lox*66
lip2t-R3	GGAGTCGCATAAGGGAGAGCTCTAGAGTCGACACGCGTTACCGTTCGTATAG CATACATTATACGAAGTTATCTCCACCTGTGTCAATCTTC	PCR for lip2t-rc*lox*71
Upleu-F1	AGCATACATTATACGAAGTTATACGCGTGCTAGCCTACGATAAGCAGTCCAA TATTCTG	PCR for Upleu-*lox*71
Upleu-R1	GGCAGGGCCCATAACTTCGTATAATGTATGCTATACGAACGGTAGACAGCA ACTACTCCTTTCAC	PCR for Upleu-*lox*71
Upleu-F2	CAAGCCCGGTCTTACGGCCATC	upstream sequence of Upleu fragment
Upleu-F3	GGTGATGGGAAGAGTCCACTCA	787-810 bp of Upleu fragment
Upleu-R3	CATACTACAATCACGAGCGCTTC	reverse complementary 820–841 bp of Upleu fragment
leu-F2	CTCATGTTTGACAGCTTATCGCTAGCTACCGTTCGTATAATGTATGCTATACG AAGTTATGATAAGCTGTCAAACATGA	PCR for rc*lox*66-partial *LEU2*
leu-R2	TCATCATTTCATTAGCAGGGCAGGGCCCTTTTTATAGAGTCTTATACAC	PCR for rc*lox*66-partial *LEU2*, *lox*71-partial *LEU2*
leu-F3	CTCATGTTTGACAGCTTATCGCTAGCTACCGTTCGTATAGCATACATTATACG AAGTTATGATAAGCTGTCAAACATGA	PCR for *lox*71-partial *LEU2*
Ura-F1	CTGTGCGGTATTTCACACCGCATATGTCAATCCAATTACCCCCCACAAC	PCR for *URA3*
Ura-R1	TTCGTCTTGTGGGATGGCATCATATGGACAAAGGCCTGTTTCTCGG	PCR for *URA3*
Apa-R3	GTACAGGTTCAGGTCCTTTCGCAGC	reverse complementary 616–641 bp of *leu2-270* and *LEU2* segments
Apa-R6	AGCGCTATCGAACGTACCCAG	reverse complementary 93–114 bp of *leu2-270* and *LEU2* segments
KNC-F2	CATCAACACCGGCCTGTGCACCTAA	3′ end sequences of *rml* gene
SXB-R2	GCGGATCCACCATTCCTTGCGGCGGCGGTGC	reverse complementary upstream sequence of hp12d promoter
qact1	CTGGCCGAGATCTTACCGAC	qPCR for *act1* gene
qact2	CATCGGGAAGCTCGTAGGAC	qPCR for *act1* gene
qRML-F	AAGACCTGGTCCACCCTGATC	qPCR for *rml* gene
qRML-F	CGGAGACAGGAGGGTAGGAA	qPCR for *rml* gene
***lox* site**		
*lox*P	ATAACTTCGTATAGCATACATTATACGAAGTTAT	wild type
*lox*71	taccgTTCGTATAGCATACATTATACGAAGTTAT	LE mutant
*lox*66	ATAACTTCGTATAGCATACATTATACGAAcggta	RE mutant
*lox*72	taccgTTCGTATAGCATACATTATACGAAcggta	LE+RE mutant

Table S2. Strains and plasmids used in this study.

Plasmid/Strain	Description	Source
**Plasmid**		
T-*cre*	pMD19T derivative, carrying *cre* gene, stored in our laboratory	
hp12d-n*rml* (n = 1–4)	expression vector carrying n (n = 1–4) copies of *rml* expression cassettes	[36]
hp12d-5*rml*	expression vector carrying 5 copies of *rml* expression cassettes	This study
hp12d-6*rml*	expression vector carrying 6 copies of *rml* expression cassettes	This study
1296-*cre*	hp12d-*rml* derivative, carrying *cre* expression cassette and two *lox*71 sites	This study
Cre-Y1	1296-*cre* derivative, carrying *URA3*	This study
hp12d-*rml-cre*	Cre-Y1 and hp12d-*rml* derivative, carrying *rml* expression cassette and a *lox*71 site	This study
hp12d-*rml-cre-lox*66	hp12d-*rml-cre* derivative, carrying a *lox*66 site	This study
Cre-Y2	hp12d-*rml-cre-lox*66 derivative, carrying a *lox*66 and a rc*lox*66 sites	This study
hp12d-*rml-cre*-rc*lox*71	hp12d-*rml-cre* derivative, carrying a rc*lox*71 site	This study
Cre-Y3	hp12d-*rml-cre-* rc*lox*71 derivative, carrying a rc*lox*71 and a *lox*71 sites	This study
pMD19-*act1*	pMD19T derivative, carrying *act1* gene	[36]
**Strain**		
W29	wild-type *Y. lipolytica* strain	
Po1f	*MatA*, *leu2-270*, *ura3-302*, *xpr2-322*, *axp1-2*, Leu^-, Ura^-, △AEP, △AXP	[39]
Po1h	*MatA*, *ura3-302*, *xpr2-322*, *axp1-2*, Ura^-, △AEP, △AXP	[40]
Po1f/Cre-Y1	Po1f derivative, carrying Cre-Y1	This study
FY1	Po1f derivative, carrying a *lox*71 site at *LEU2* gene locus	This study
FY1/Cre-Y2	FY1 derivative, carrying Cre-Y2	This study
FY2-*rml*	FY1 derivative, carrying a *rml* expression cassette	This study
FY2-rc*rml*	FY1 derivative, carrying a rc*rml* expression cassette	This study
FY2-*rml*/Cre-Y3	FY2-*rml* derivative, carrying Cre-Y3	This study
FY3-2*rml*	FY2-*rml* derivative, carrying 2 copies of *rml* expression cassettes	This study
FY3-*rml*-rc*rml*	FY2-*rml* derivative, carrying a *rml* and a rc*rml* expression cassettes	This study
FY4-3*rml*	FY3-2*rml* derivative, carrying 3 copies of *rml* expression cassettes	This study
FY4-2*rml*-rc*rml*	FY3-2*rml* derivative, carrying 2 copies *rml* a rc*rml* expression cassettes	This study
FY5-4*rml*	FY4-3*rml* derivative, carrying 4 copies of *rml* expression cassettes	This study
FY5-3*rml*-rc*rml*	FY4-3*rml* derivative, carrying 3 copies of *rml* and a rc*rml* expression cassettes	This study
FY6-5*rml*	FY5-4*rml* derivative, carrying 5 copies of *rml* expression cassettes	This study
FY6-4*rml*-rc*rml*	FY5-4*rml* derivative, carrying 4 copies of *rml* and a rc*rml* expression cassettes	This study
FY7-6*rml*	FY6-5*rml* derivative, carrying 6 copies *rml* expression cassettes	This study
FY7-5*rml*-rc*rml*	FY6-5*rml* derivative, carrying 5 copies of *rml* and a rc*rml* expression cassettes	This study
Po1f/hp12d-n*rml*	Po1f derivative, carrying n copies of *rml* expression cassettes (n = 1–6)	This study

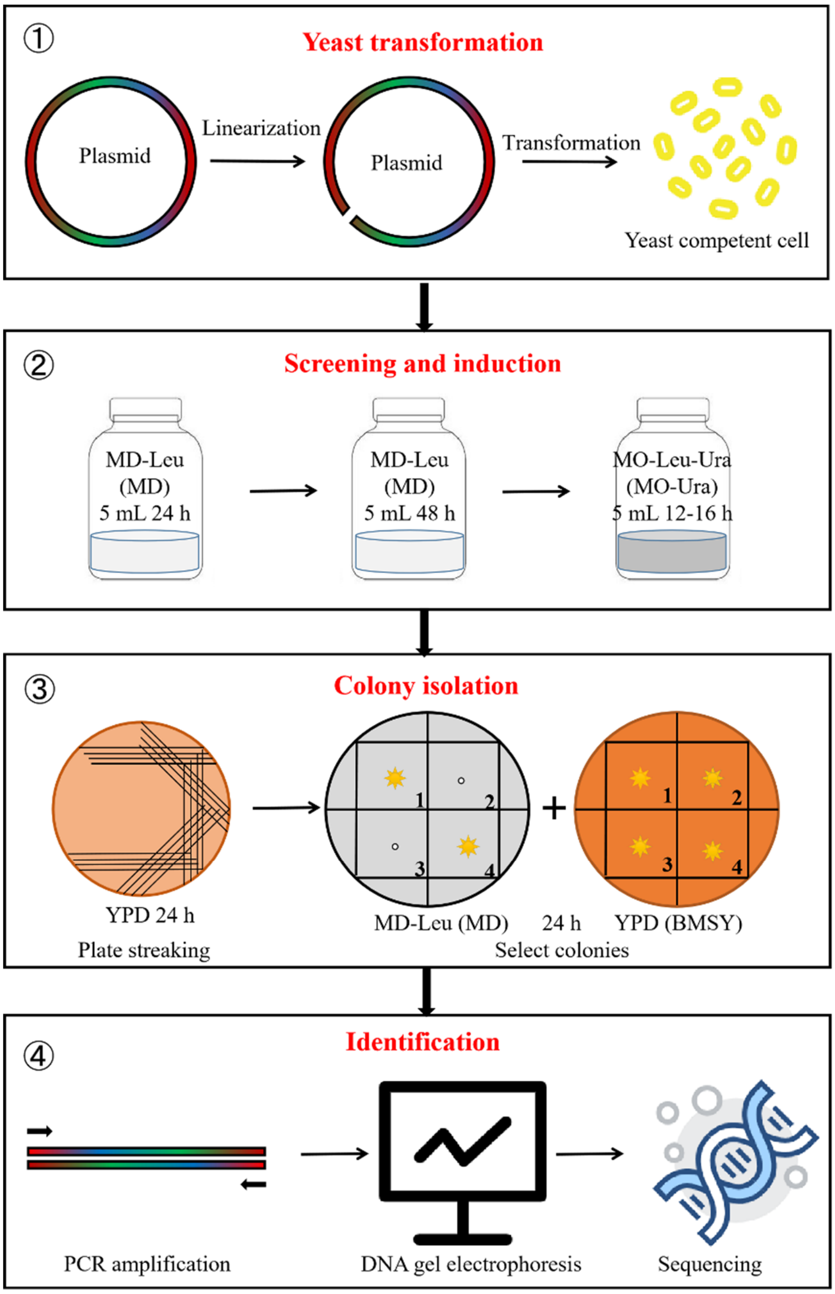

Figure S1. Illustration of the gene integration process employed in this study. The integration process included yeast transformation, screening and induction, colony isolation, and identification. First, the plasmids were linearized and used to transform yeast competent cells. Then, the transformants were screened in 5 mL of MD-Leu (MD) medium for 24 h, and rescreened in 5 mL of MD-Leu (MD) liquid medium for 48 h. Subsequently, 100 μL of the cell suspension solutions were induced in 5 mL of MO-Leu-Ura (MO-Ura) liquid medium. Afterwards, single colony was obtained by plate streaking on YPD medium and phenotype identification on YPD (BMSY) and MD-Leu (MD) media. The selected positive colonies were further confirmed via PCR amplification and DNA sequencing.

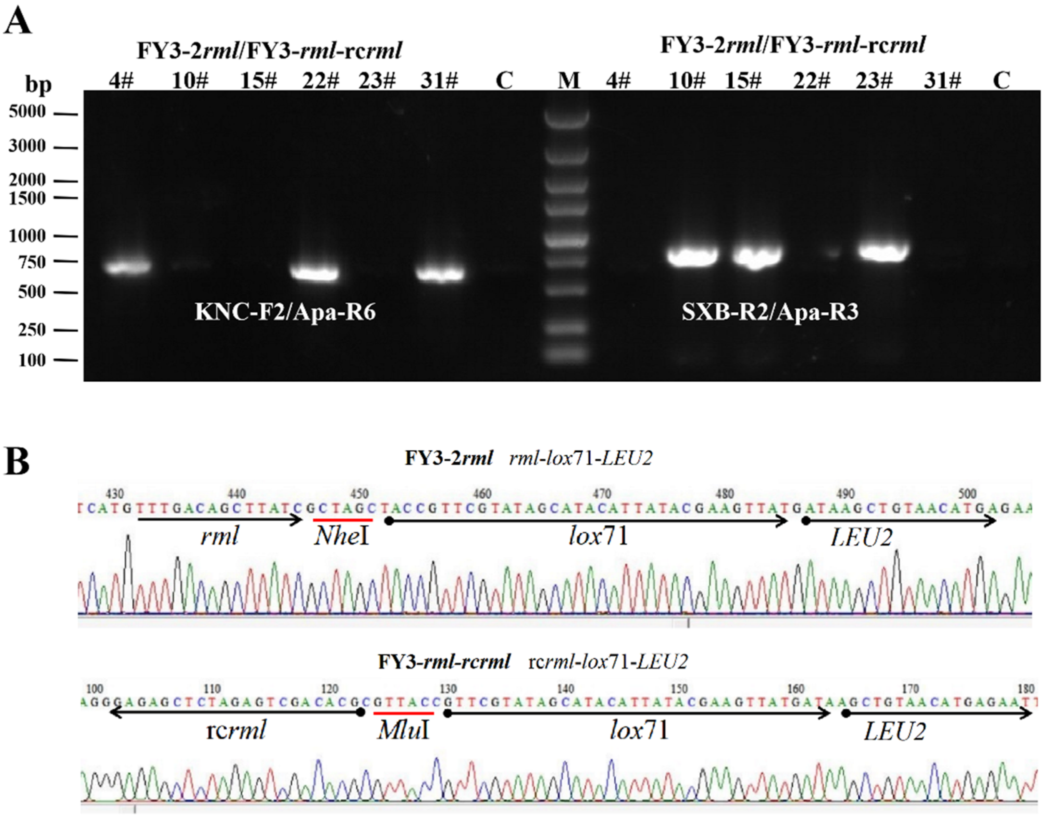

Figure S2. (**A**) PCR amplification to identify positive colonies in the third-round integration. Lane M, Marker; lane C, ddH$_2$O. PCR products of about 650 bp (corresponding to partial *rml*-rc*lox*66/*lox*71-partial *LEU2*) were amplified with primer pair KNC-F2/Apa-R6 only in FY3-2*rml*, and DNA fragments of about 800 bp (corresponding to partial rc*rml*-rc*lox*66/*lox*71-partial *LEU2*) were cloned using SXB-R2/Apa-R3 in FY3-*rml*-rc*rml*. (**B**) Partial sequencing result of PCR products *rml*-*lox*71-*LEU2* from FY3-2*rml* and rc*rml*-*lox*71-*LEU2* from FY3-*rml*-rc*rml*.

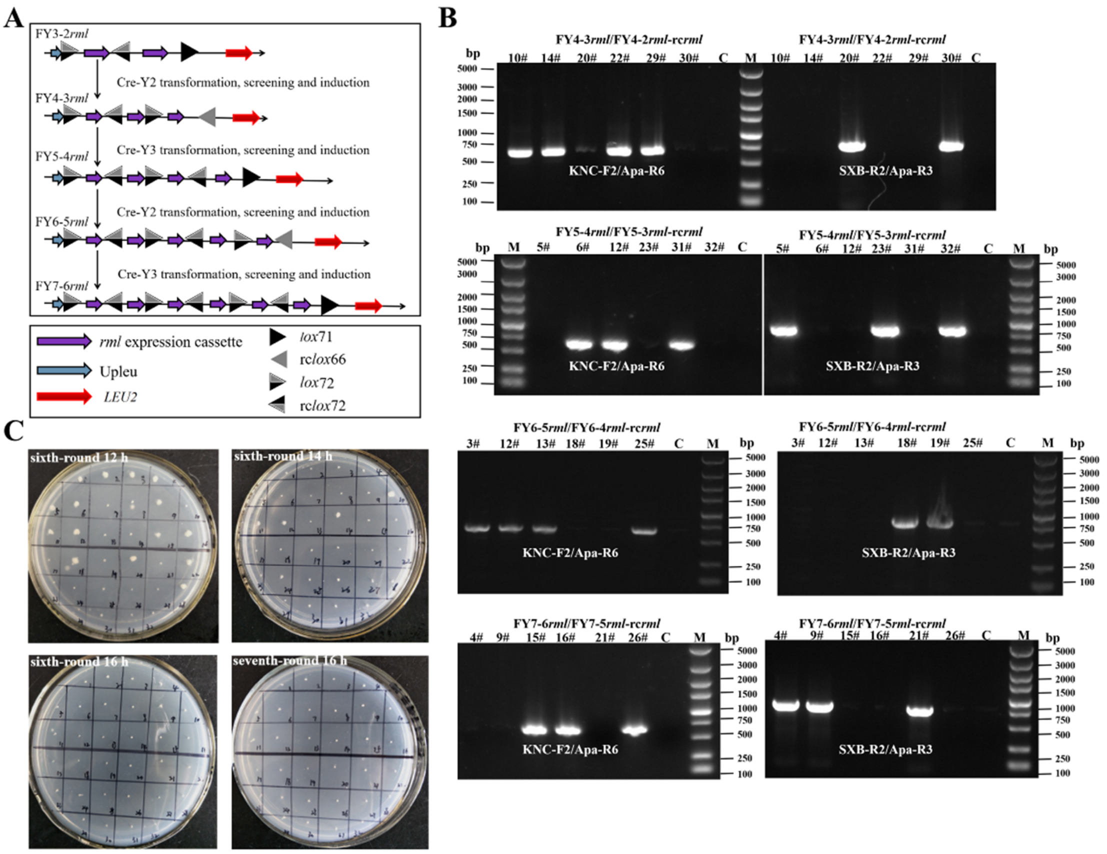

Figure S3. (**A**) Schematic diagram of the fourth–seventh rounds of integration. The recombinant strains harboring rc*rml* expression cassettes were not present. (**B**) PCR amplification to identify positive colonies in the fourth–seventh rounds of integration. The PCR products on the left (about 650 bp, corresponding to partial *rml*-rc*lox*66/*lox*71-partial *LEU2*) were amplified with primer pair KNC-F2/Apa-R6, and the DNA fragments on the right (about 800 bp, corresponding to partial rc*rml*-rc*lox*66/*lox*71-partial *LEU2*) were cloned using SXB-R2/Apa-R3. Lane M, Marker; lane C, ddH$_2$O. (**C**) Positive colonies induced for 12–16 h in the sixth and seventh rounds of integration. The corresponding BMSY plates are not shown.

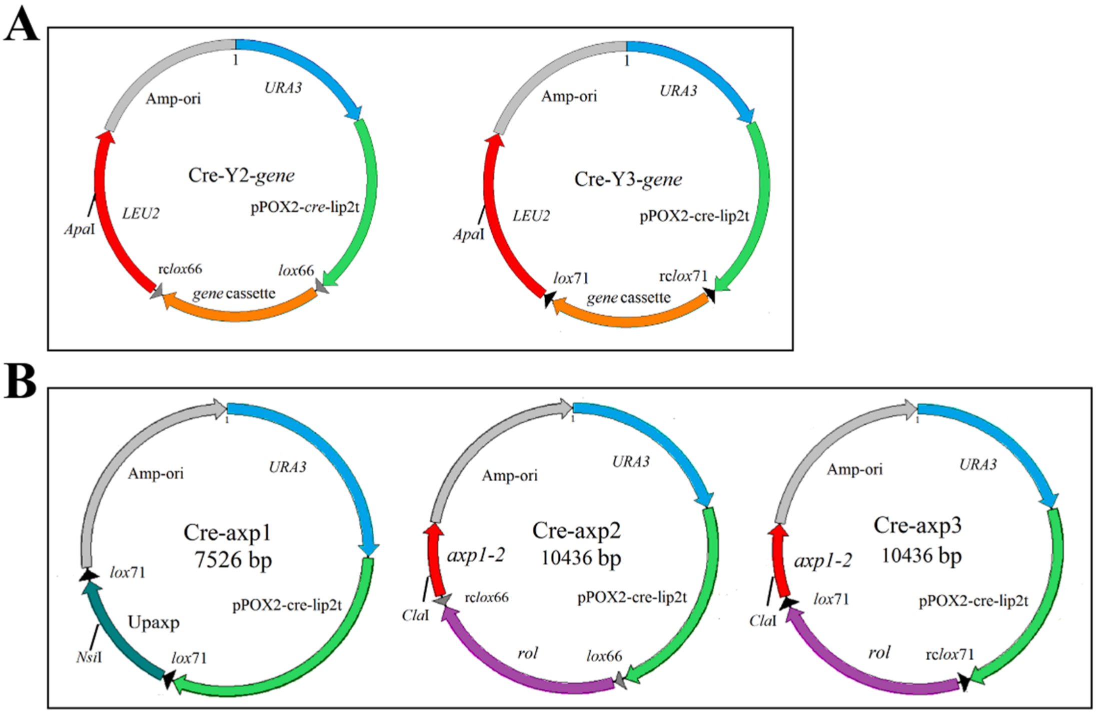

Figure S4. (**A**) Plasmids for various gene integrations in *LEU2* locus of Po1f. These plasmids were derived from Cre-Y2 or Cre-Y3 and carried one of the following genes: *ire1*, *kar2*, *pdi*, *sls1*, *hac1*, and *vgb*. All plasmids were linearized by *Apa*I at homologous fragment *LEU2* and transformed into the engineered strain FY5-4*rml* or its derived strains. The MD, MO-Ura and BMSY media were used for screening, induction and colony isolation, and the primer pairs KNC-F3 (CCTACTACCTGTCCATCAATG)/Apa-R6 and SXB-R2/Apa-R3 were applied for strain identification. (**B**) Plasmids for *rol* gene integration in *axp1-2* locus of Po1h. The plasmids Cre-axp1, Cre-axp2, and Cre-axp3 were respectively derived from Cre-Y1, Cre-Y2, and Cre-Y3, and their integration processes were also similar. The Upaxp is the upstream segment of *axp1-2* gene (1059 bp) and *axp1-2* is designed as the homologous fragment of *axp1-2* in Po1h, and their functions are the same as Upleu and *LEU2* in the original genetic tool.

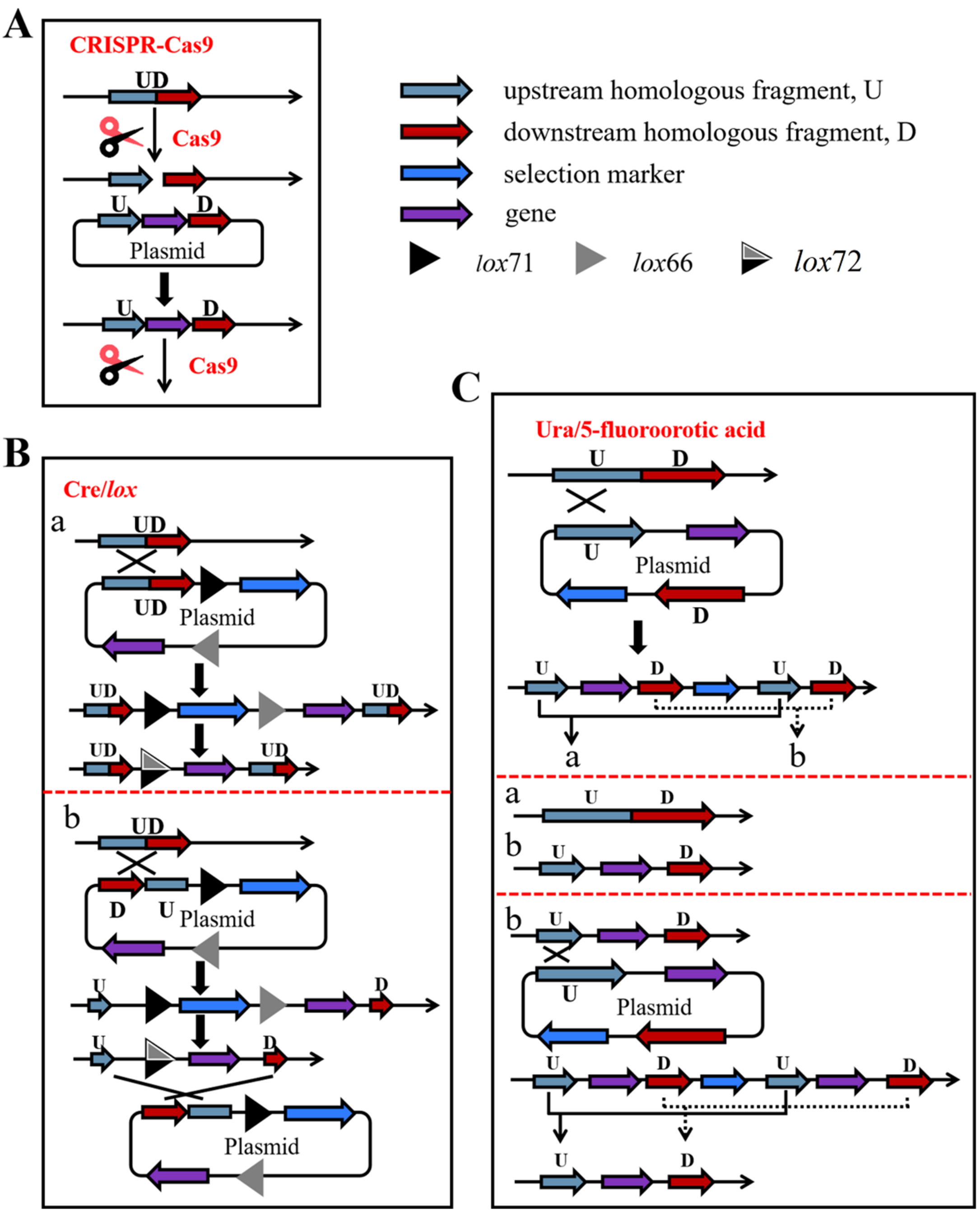

Figure S5. Repeated, targeted and markerless gene integration via the existing genetic tools based on CRISPR-Cas9 system, Cre/*lox* system, and Ura/5-fluoroorotic acid resistance method. The integrated states are similar if the plasmids in this figure are replaced by DNA fragments carrying target genes. (**A**) Cas9 protein and sgRNA can cause double-strand break (DSB) in the homologous fragment UD, and the target gene can be integrated into UD locus via HR repair. However, the target gene may be broken if the same sgRNA is used for subsequent gene integration. Thus, more integration rounds require additional plasmids harboring new sgRNAs. (**B**) Recombinant plasmid carrying target gene is integrated into homologous fragment UD via gene insertion (a) or gene replacement (b), and the selection marker can be removed by deletion reaction between *lox* sites. Gene insertion event is highly frequent and can retain two UD fragments, resulting in gene redundancy in the genome. On the contrary, gene replacement event is more suitable, but infrequent. Nonetheless, target gene will be replaced if the UD fragment is used for second-round integration. Therefore, further integration requires different homologous fragments. (**C**) Plasmid harboring target gene is inserted into the homologous fragment U. Although two kinds of recombination reactions (a and b) may occur via Ura/5-fluoroorotic acid resistance, only reaction b produces target strain. Moreover, the recombination reactions will generate reversible strains when the same plasmid is integrated in the above-mentioned target strain (the HR of target gene segments is not present). Hence, new homologous fragments should be employed for additional gene integration.

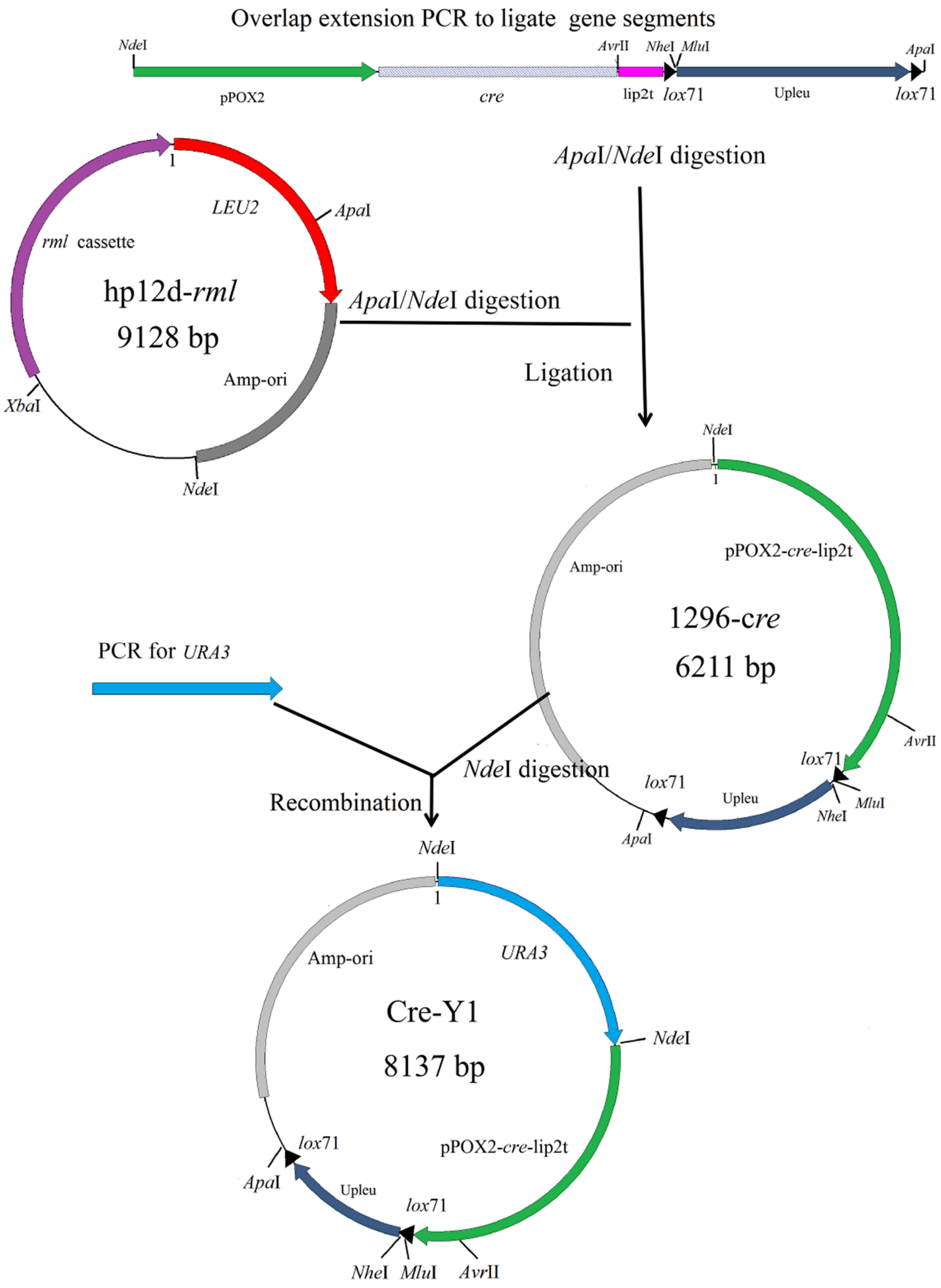

Figure S6. Process diagram for constructing plasmid Cre-Y1.

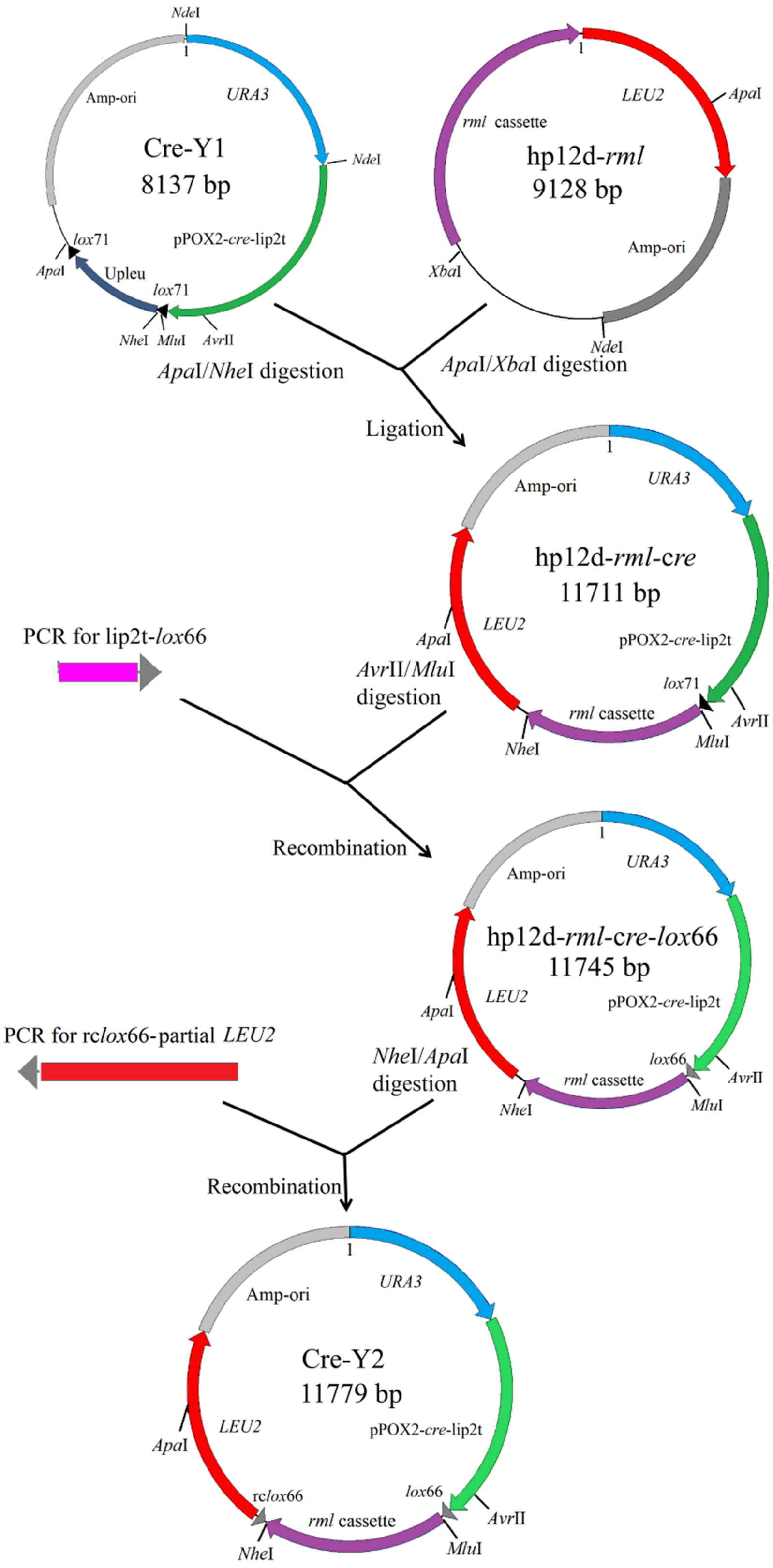

Figure S7. Process diagram for constructing plasmid Cre-Y2.

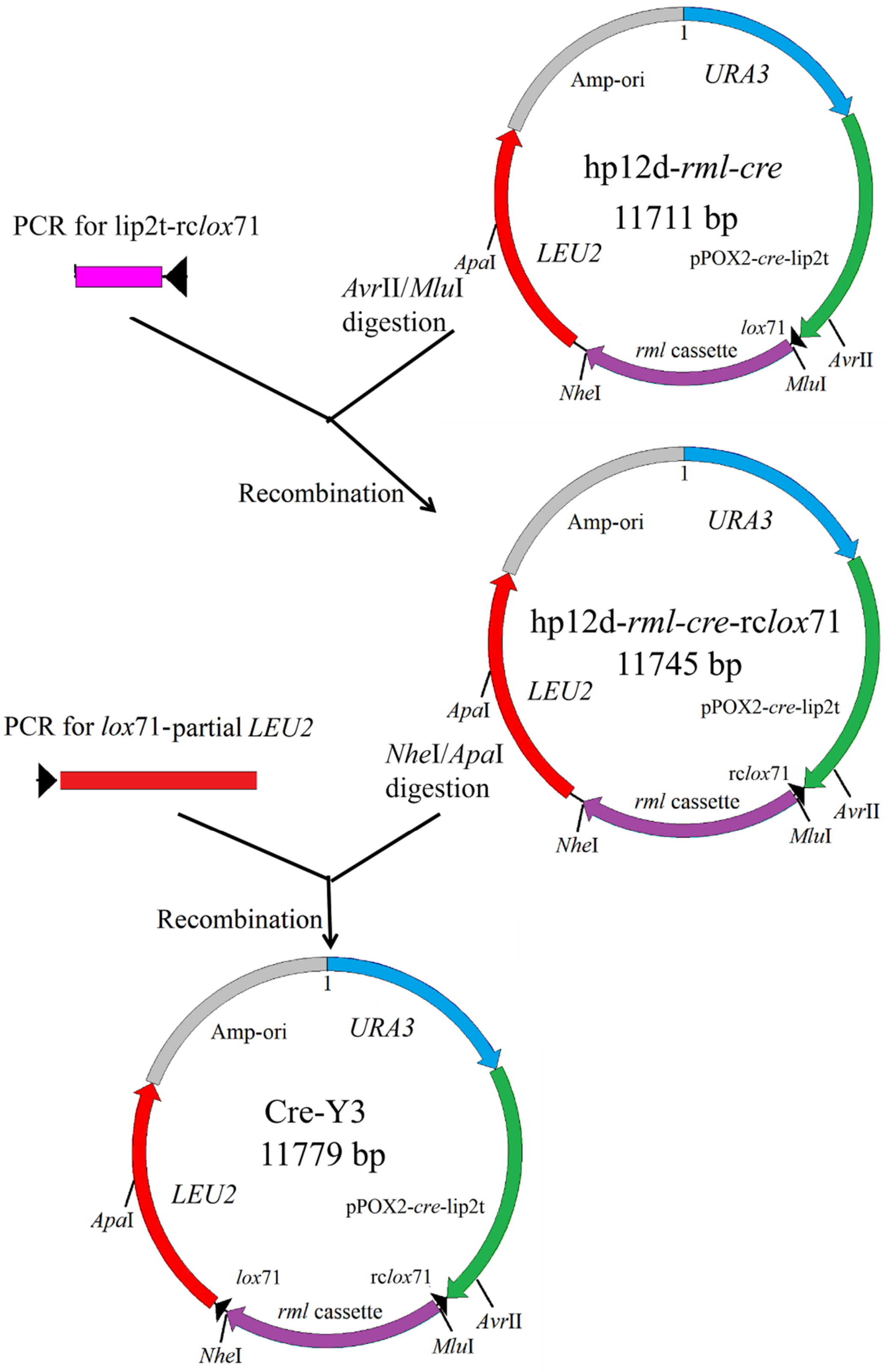

Figure S8. Process diagram for constructing plasmid Cre-Y3.
